# The Printability, Microstructure, and Mechanical Properties of Fe_80−*x*_Mn*_x_*Co_10_Cr_10_ High-Entropy Alloys Fabricated by Laser Powder Bed Fusion Additive Manufacturing

**DOI:** 10.3390/mi15010123

**Published:** 2024-01-11

**Authors:** Kai Li, Vyacheslav Trofimov, Changjun Han, Gaoling Hu, Zhi Dong, Yujin Zou, Zaichi Wang, Fubao Yan, Zhiqiang Fu, Yongqiang Yang

**Affiliations:** School of Mechanical and Automotive Engineering, South China University of Technology, Guangzhou 510641, China; 202120100662@mail.scut.edu.cn (K.L.); trofimov@scut.edu.cn (V.T.); menhgling@mail.scut.edu.cn (G.H.); medongzhi@mail.scut.edu.cn (Z.D.); 202220100495@mail.scut.edu.cn (Y.Z.); 202220100483@mail.scut.edu.cn (Z.W.); mefbyan@mail.scut.edu.cn (F.Y.); meyqyang@scut.edu.cn (Y.Y.)

**Keywords:** additive manufacturing, high-entropy alloys, FeMnCoCr, laser powder bed fusion, transformation-induced plasticity

## Abstract

This work investigated the effect of Fe/Mn ratio on the microstructure and mechanical properties of non-equimolar Fe_80−*x*_Mn*_x_*Co_10_Cr_10_ (*x* = 30% and 50%) high-entropy alloys (HEAs) fabricated by laser powder bed fusion (LPBF) additive manufacturing. Process optimization was conducted to achieve fully dense Fe_30_Mn_50_Co_10_Cr_10_ and Fe_50_Mn_30_Co_10_Cr_10_ HEAs using a volumetric energy density of 105.82 J·mm^−3^. The LPBF-printed Fe_30_Mn_50_Co_10_Cr_10_ HEA exhibited a single face-centered cubic (FCC) phase, while the Fe_50_Mn_30_Co_10_Cr_10_ HEA featured a hexagonal close-packed (HCP) phase within the FCC matrix. Notably, the fraction of HCP phase in the Fe_50_Mn_30_Co_10_Cr_10_ HEAs increased from 0.94 to 28.10%, with the deformation strain ranging from 0 to 20%. The single-phase Fe_30_Mn_50_Co_10_Cr_10_ HEA demonstrated a remarkable combination of high yield strength (580.65 MPa) and elongation (32.5%), which surpassed those achieved in the FeMnCoCr HEA system. Comparatively, the dual-phase Fe_50_Mn_30_Co_10_Cr_10_ HEA exhibited inferior yield strength (487.60 MPa) and elongation (22.3%). However, it displayed superior ultimate tensile strength (744.90 MPa) compared to that in the Fe_30_Mn_50_Co_10_Cr_10_ HEA (687.70 MPa). The presence of FCC/HCP interfaces obtained in the Fe_50_Mn_30_Co_10_Cr_10_ HEA resulted in stress concentration and crack expansion, thereby leading to reduced ductility but enhanced resistance against grain slip deformation. Consequently, these interfaces facilitated an earlier attainment of yield limit point and contributed to increased ultimate tensile strength in the Fe_50_Mn_30_Co_10_Cr_10_ HEA. These findings provide valuable insights into the microstructure evolution and mechanical behavior of LPBF-printed metastable FeMnCoCr HEAs.

## 1. Introduction

Laser powder bed fusion (LPBF) is a disruptive additive manufacturing (AM) technology with the potential to revolutionize the fabrication of geometrically complex products from metal powders, overcoming the limitations of conventional processing methods [1,2]. Its ultra-high cooling rate of 10^3^–10^8^ K/s during non-equilibrium solidification effectively inhibits grain growth and the segregation of alloying elements within metal materials, which are challenging to achieve through conventional processing methods [3]. LPBF enables the production of metallic materials with microstructures characterized by highly heterogeneous grain geometries, sub-grain dislocation structures, and chemical segregation [4,5], thus resulting in exceptional mechanical properties.

High-entropy alloys (HEAs) have recently emerged as a novel paradigm for designing multi-principle-element alloys and attract significant attention from both academia and industry due to their distinctive microstructures and exceptional structural-functional properties [6]. According to phase types, HEAs can be generally categorized into single face-centered cubic (FCC) phase, such as the CoCrFeMnNi system (also known as Cantor alloys [7]); body-centered cubic (BCC) phase, such as the refractory NbMoTaW system; dual-phase such as FCC + BCC; or hexagonal close-packed (HCP) phase based on the structure of the phases [8]. Through optimizing the combination of constituent elements and modulating their proportions, HEAs can exhibit excellent mechanical properties. However, most of the reported studies have focused on conventional processes used for processing HEAs such as FeMnCoCrNi [9,10,11,12,13,14] and FeMnCoCr [15,16,17,18], resulting in inadequate yield strengths that limit practical engineering applications including jet aircraft turbine blades, nuclear fusion reactors, etc. Fortunately, LPBF offers rapid melting and solidification rates that promote the formation of refined grains [19], segregated phases [20], and high-density dislocation [21], which play a crucial role in strengthening the material by regulating dislocation motion during deformation.

The primary strengthening mechanisms of single-phase HEAs are solid-solution strengthening and dislocation strengthening, which enhance strength at the expense of plasticity. However, with the development of HEAs, design concepts based on equimolar ratios and single-phase microstructures have become limited. Therefore, the concept of “metastability-engineering” has been introduced in certain non-equimolar HEAs. Li et al. further expanded this concept by adjusting the Mn content in metastable FeMnCoCr HEAs to modulate the stacking fault energy of the matrix phase, thereby increasing the driving force (ΔG) for austenite-to-martensite (γ→ε) transformation within the matrix [16]. By promoting a dual-phase microstructure through composition design in FeMnCoCr HEAs, various mechanisms such as transformation-induced plasticity (TRIP), solid-solution lattice distortion, and dislocations can be utilized to strengthen their mechanical properties and overcome trade-offs between strength and ductility [22,23].

Engineering the martensitic transformation or TRIP effect has been validated as a viable and effective method of achieving superior strength–ductility synergy. Antolovich et al. reported that austenite-to-martensite at the crack of TRIP steels could assimilate additional energy [24]. Agrawal et al. demonstrated that the martensitic transformation delayed the crack initiation and prolonged crack extension life in the high cycle fatigue response of Fe_40_Mn_20_Co_20_Cr_15_Si_5_ HEA fabricated via LPBF [25]. Wang et al. discovered that the generation of compressive stress during martensitic transformation inhibited crack initiation [26]. However, some studies have shown that the transformation effect may degrade the ductility of alloys [27,28,29,30]. Unfortunately, the optimal combination of ductility and strength in HEAs remains poorly understood, due to potential deformation-induced transformation probably compromising their mechanical properties. Additionally, it has been observed that such transformation not only enhance plasticity but also induce local brittleness and nucleation damages [29]. Consequently, significant efforts were made to fabricate high-performance FeMnCoCr HEAs via LPBF while further exploring the mechanisms of martensitic transformation.

The FeMnCoCr high-entropy alloy system exhibits excellent mechanical properties overcoming the strength-ductility trade-off. Moreover, the Mn content plays an important role in the phase composition and phase stability of the alloy system. Specifically, the Mn content of 40 at% represents a critical point for the transition in deformation mechanisms, shifting from dislocation-dominated plasticity to twinning-induced plasticity and transformation-induced plasticity [16]. Therefore, Fe_30_Mn_50_Co_10_Cr_10_ (with a Mn content of 50 at%) and Fe_50_Mn_30_Co_10_Cr_10_ (with a Mn content of 30 at%) were selected for further investigation. However, at present, while a large amount of progress has been made towards LPBF-printed single-phase system HEAs, few studies have been carried out on single- and dual-phase FeMnCoCr systems. Furthermore, this is the first simultaneous study of the additive manufacturing of stable and metastable non-equimolar FeMnCoCr HEAs by tuning Mn elemental composition. This study investigated the processing optimization, microstructure, and mechanical properties of Fe_80−*x*_Mn*_x_*Co_10_Cr_10_ HEAs fabricated by LPBF. Specifically, the effect of process parameters as well as Fe/Mn contents on the printability, microstructure, and mechanical properties of the LPBF-printed Fe_80−*x*_Mn*_x_*Co_10_Cr_10_ HEAs were analyzed. A comparison of the microstructure and mechanical properties between Fe_30_Mn_50_Co_10_Cr_10_ and Fe_50_Mn_30_Co_10_Cr_10_ HEAs is discussed, while deformation-strengthening damage mechanisms are further elucidated. The underlying mechanisms behind TRIP effect and strengthening behavior during tensile deformation are revealed.

## 2. Materials and Experiments

### 2.1. Materials

**Figure 1:**
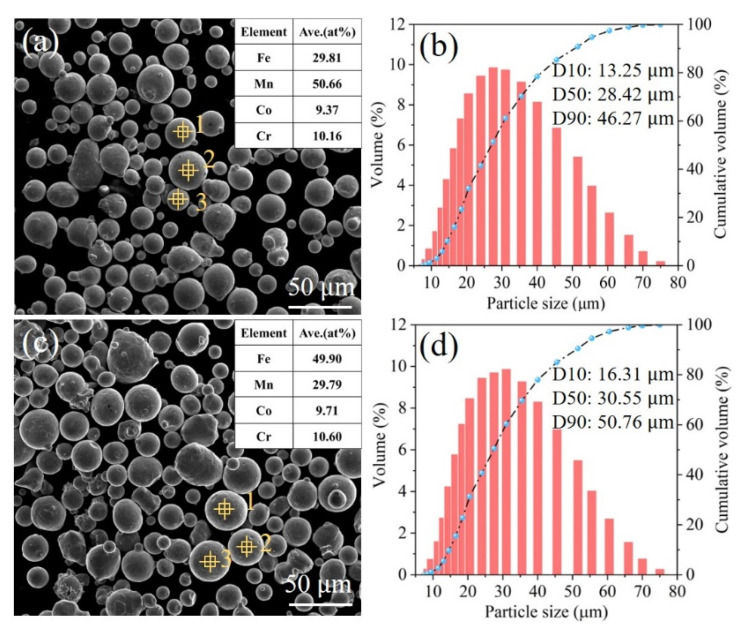
Characteristics of the Fe_80−*x*_Mn*_x_*Co_10_Cr_10_ powders: (**a**,**c**) SEM images showing the morphology of the Fe_30_Mn_50_Co_10_Cr_10_ and Fe_50_Mn_30_Co_10_Cr_10_ powders, respectively, and inserted tables indicate the composition of the powders, (**b**,**d**) particle size distribution of the powders, respectively. Numbers 1, 2, and 3 represent EDS spectral point measurements.

The pre-alloyed HEA powders used were Fe_30_Mn_50_Co_10_Cr_10_ (at%) and Fe_50_Mn_30_Co_10_Cr_10_ (at%) with a particle size distribution from 5 to 75 μm, which exhibited a stabilized single FCC phase and a metastable dual phase, respectively. The morphologies and element distributions of the powders were detected using a scanning electron microscope (SEM, FEI Quanta 250, Eindhoven, The Netherlands) with energy-dispersive spectroscopy (EDS) analysis. Both powder particles displayed near-spherical shapes (Figure 1a,c), as shown in the inserted images depicting the measured compositions of the powders, while three randomly selected powder particles were subjected to compositional analysis, indicating homogeneous elemental distribution. The average particle size D50 of the powders, measured using a laser particle size analyzer, increased from 28 to 30 μm upon decreasing the Mn content from 30 to 50 at% (Figure 1b,d).

### 2.2. Sample Fabrication by LPBF

**Figure 2:**
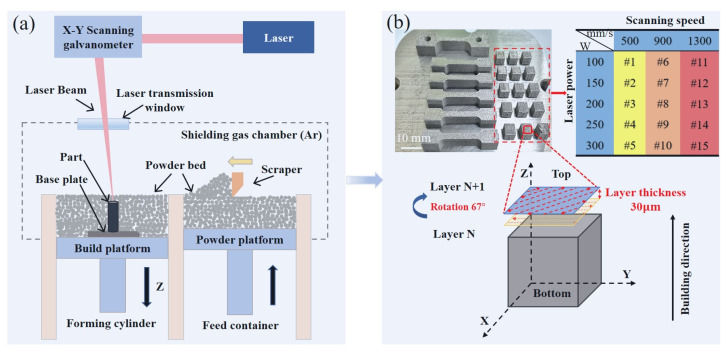
(**a**) Schematic illustration of the LPBF process, and (**b**) printed HEA samples and process parameters used.

The LPBF process was conducted using a Dimetal-100 machine (Laseradd Technology, Guangzhou, China) with a 1064 nm Nd: YAG fiber laser and a two-axis scanner. A schematic illustration of the HEA samples produced by LPBF is shown in Figure 2a. The two types of Fe_80−*x*_Mn*_x_*Co_10_Cr_10_ samples were printed on 316L stainless steel substrates and subsequently separated from the substrates by electrical discharge machining. The samples with sizes of 6 × 6 × 6 mm^3^ were printed using various combinations of process parameters as shown in Figure 2b, which included laser power (*P*) ranging from 100 W to 300 W, scanning speed (*v*) ranging from 500 mm/s to 1300 mm/s, hatch space (*h*) of 70 μm, a layer thickness (*t*) of 30 μm, and a scanning strategy involving rotating neighboring layers by 67° that can effectively mitigate residual stresses, thereby minimizing the risk of cracking in the fabricated samples. Volumetric energy densities (VEDs) were evaluated based on these parameters, i.e., VED = *P*/(*vht*). The same VEDs were used to print Fe_30_Mn_50_Co_10_Cr_10_ and Fe_50_Mn_30_Co_10_Cr_10_ HEAs for comparing their printability, microstructural evolution, and mechanical properties. The dog bone-shaped samples measuring 42 × 9 × 6 mm^3^ using different VEDs were fabricated and subsequently cut into tensile samples with dimensions of 42 × 9 × 1.5 mm^3^.

### 2.3. Characterizations

The relative density of the LPBF-printed HEAs depended on the ratio of the experimental density determined using Archimedes’ principle to the theoretical density, and each sample was tested three times to ensure that the results were reproducible. For microstructural characterizations, the samples were sequentially polished with 200-, 400-, 800-, 1200-, 2000-, and 3000-grit SiC sandpaper, and then polished using 2 μm sized diamond polishing particles until the surfaces were smooth without any scratches, and the polished sample surfaces were etched with aqua regia solution (HNO_3_:HCl = 1:3) for 30 s. The phase identification of the HEAs was performed via X-ray diffraction (XRD, Malvern Panalytical, Shanghai, China). The surface morphologies were characterized using an optical microscope (OM, Zeiss, Munich, Germany) and SEM, and composition characterization was conducted using an SEM equipped with an energy-dispersive spectrometer. The samples were polished by mechanical vibration on an electron-backscattered diffraction apparatus (EBSD, FEI Nova NanoSEM230, Hillsboro, OR, USA) for testing the microstructures of kernel average misorientation (KAM), inverse polar figure (IPF), and phase diagram used for analyzing the results with channel5 software.

### 2.4. Mechanical Testing

In microhardness testing, the indentation of the sample was performed on a digital microhardness tester (MVS-1000D1, Shanghai, China) with a Vickers indenter, applying a load of 0.2 kg for 15 s and ensuring the uniform distribution of five test points on the samples. The universal testing machine (SUST CMT5504, Zhuhai, China) was used to explore the tensile properties of the HEA samples, with a loading speed of 1 mm/min and a mechanical extensometer with a gauge length of 10 mm. Three samples were tested at room temperature for each printed HEA to verify the reproducibility of the mechanical properties, such as its yield strength (σ_y_), ultimate tensile strength (σ_u_), and elongation (δ). Intermittent tensile tests at strains of 10% and 20% were performed on the same samples, followed by cutting the fractured center region for an analysis of the deformed microstructure. The fracture morphology of the tensile samples was determined using a scanning electron microscope.

## 3. Results and Discussion

### 3.1. The Printability of LPBF-Printed Fe_80−x_Mn_x_Co_10_Cr_10_ HEAs

**Figure 3:**
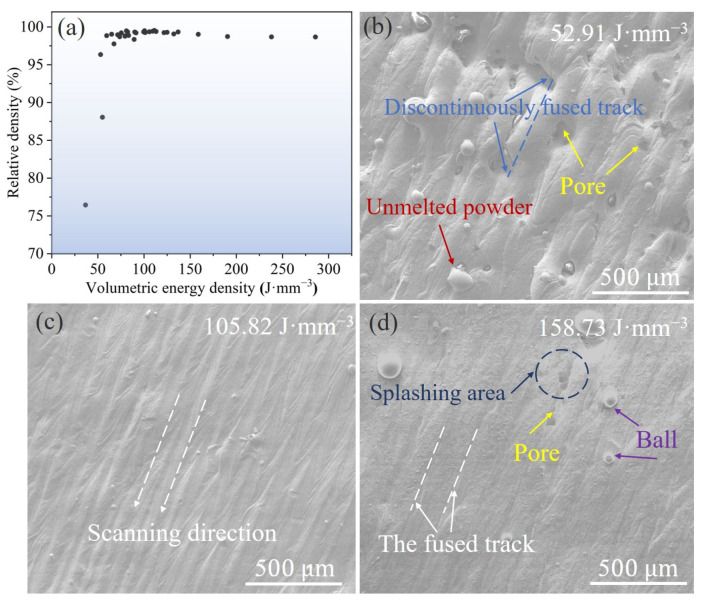
(**a**) Relative density of as-printed Fe_30_Mn_50_Co_10_Cr_10_ HEA samples with various volumetric energy densities. SEM images exhibiting the representative surface morphology of the as-printed samples under different VEDs: (**b**) 52.91 J·mm^−3^, (**c**) 105.82 J·mm^−3^, and (**d**) 158.73 J·mm^−3^.

The relative density of the Fe_30_Mn_50_Co_10_Cr_10_ HEA samples exhibited an initial increase followed by a decrease as the VED increased from 36.63 to 285.71 J·mm^−3^ (Figure 3a). Notably, the relative density remained above 99% within a VED range of 75~150 J·mm^−3^. However, a slight decline in the relative density was observed with an increase in the VED, which can be attributed to the formation of pores in the samples under high-VED conditions. The augmentation of the Marangoni effect and elemental evaporation at elevated VEDs leads to increased porosity and a resultant reduction in relative density. Figure 3b–d illustrate the typical surface morphology of the printed samples. In the case of a VED of 52.91 J·mm^−3^, unmelted powder particles and pores were discernible on the sample surface along with discontinuities in melt track formation. With the VED increasing to 105.82 J·mm^−3^, a flat surface was achieved with continuous melt track formation, resulting in improved printing quality. However, at higher VEDs, overlapping melt tracks became evident along with pore formation on the sample surface and an increase in splashing phenomena [31].

**Figure 4:**
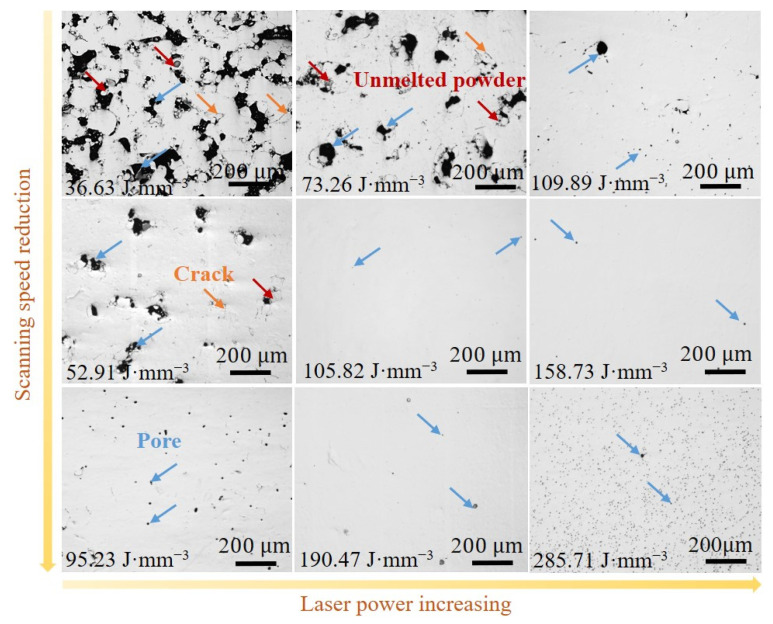
Optical microscopic images of the LPBF-printed Fe_30_Mn_50_Co_10_Cr_10_ HEAs with different VEDs. The red, blue and orange arrows represent unmelt powder, pore and crack, respectively.

Figure 4 presents optical microscope images of the LPBF-printed Fe_30_Mn_50_Co_10_Cr_10_ samples with the combination of laser power and scan speed. The surface morphology of the printed samples exhibits defects such as unmelted powder particles, cracks, and pores. Initially, an increase in VED led to a decrease in the number of defects before eventually increasing, which is consistent with the trend observed for relative density. At high VEDs (above 190 J·mm^−3^), numerous small circular pores appeared on the sample surface due to the low boiling point of Mn (2324 °C) and the smallest heat of vaporization (220.7 kJ/mol), making it susceptible to vaporization under higher laser energy densities. Consequently, this results in intensified recoil pressure within the melt pool, promoting the formation of pores that are prone to axial fluctuations and radial perturbations governed by energy and pressure balances [32]. Moreover, this phenomenon induces instability and poses a risk of pore collapse, which can lead to gas bubble formation within the melt pool that may become trapped by a solidification front [33]. These small pores act as sources for stress concentration while also serving as locations for crack initiation and propagation that can degrade mechanical properties.

**Figure 5:**
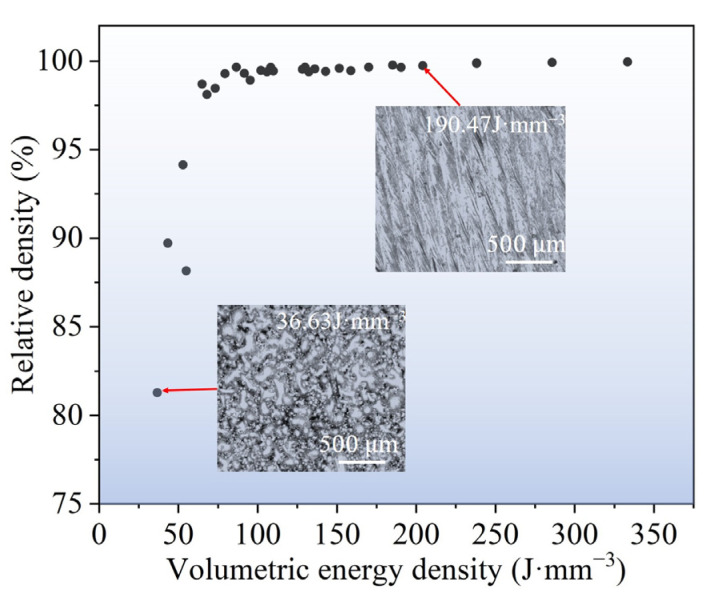
Relative density of as-printed Fe_50_Mn_30_Co_10_Cr_10_ HEA samples, and inserted image presents representative surface morphology of as-printed samples under different VEDs.

The volumetric energy density increased from 36.63 to 333.33 J·mm^−3^ (Figure 5), with an increase in the relative density of the LPBF-printed Fe_50_Mn_30_Co_10_Cr_10_ HEAs. Particularly, relative densities exceeding 99% were achieved at VEDs above 85 J·mm^−3^. Figure 5 demonstrates that at a VED of 36.63 J·mm^−3^, numerous unmelted powder particles and pores are observed on the sample surface, while discontinuous melt tracks are formed. However, when the VED reaches 190.47 J·mm^−3^, a flat surface morphology with visible melt tracks devoid of significant defects is attained.

**Figure 6:**
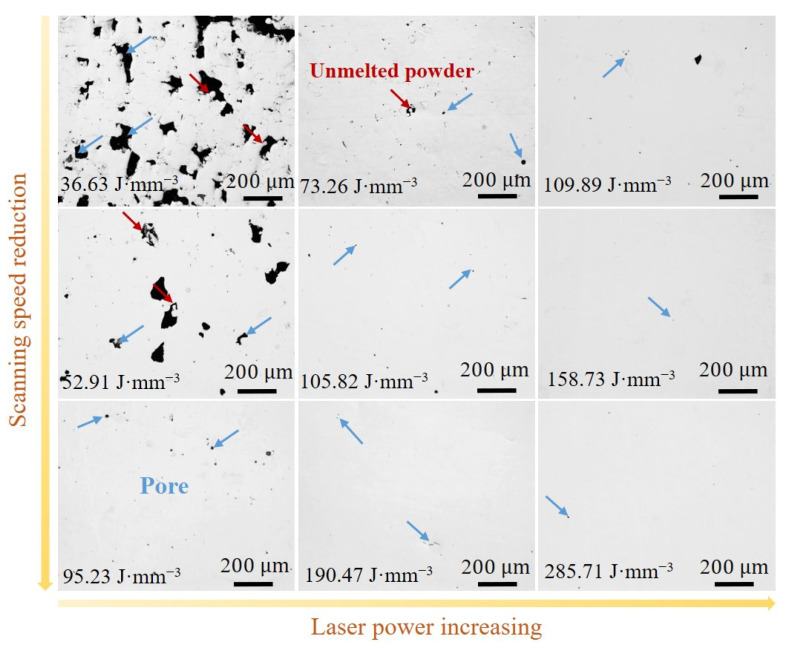
Optical microscopic images of the LPBF-printed Fe_50_Mn_30_Co_10_Cr_10_ HEA samples processed with different VEDs. The red and blue arrows represent unmelt powder and pore, respectively.

The surface morphology of LPBF-printed Fe_50_Mn_30_Co_10_Cr_10_ HEA samples with various laser powers and scanning speeds is depicted in Figure 6. The results reveal that the printed samples primarily exhibited defects including unmelted powder particles and pores. An inverse relationship between defects and VED is observable, which aligns to the trend in the relative density of the HEA samples. In comparison to Fe_30_Mn_50_Co_10_Cr_10_, the Fe_50_Mn_30_Co_10_Cr_10_ samples printed at high VEDs (above 190 J·mm^−3^) displayed fewer round pores on their surfaces. This can be attributed to the higher content of Fe, which possesses a high melting and boiling point compared to Mn, thereby promoting homogeneity in heat conduction at the two-phase interface during powder melting and the uniform distribution of elements within the melt pool. These factors contribute to reducing temperature gradients and solute solubility differences across different regions of the melt pool, inhibiting Marangoni effect formation [34]. Consequently, there were minimal defects on the surface of the Fe_50_Mn_30_Co_10_Cr_10_ samples under high-VED conditions.

### 3.2. The Microstructure of LPBF-Printed Fe_80−x_Mn_x_Co_10_Cr_10_ HEAs

**Figure 7:**
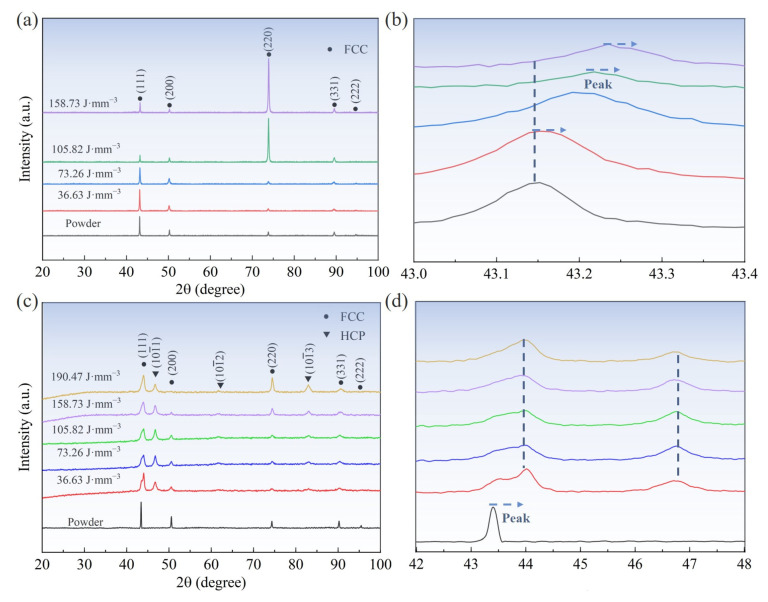
XRD patterns of the Fe_80−*x*_Mn*_x_*Co_10_Cr_10_ powders and their LPBF-printed samples: (**a**) different VEDs of Fe_30_Mn_50_Co_10_Cr_10_, (**b**) enlarged drawing of peak (111) in (**a**), (**c**) different VEDs of Fe_50_Mn_30_Co_10_Cr_10_, (**d**) enlarged drawing of peak (111) in (**c**).

The Fe_30_Mn_50_Co_10_Cr_10_ HEA powders and their LPBF-printed samples exhibit a single-phase austenitic crystal structure (Figure 7a). The shift towards higher diffraction angles for peak (111), which changed from 43.15° to 43.25°, indicates a decrease in lattice parameters within the HEA as the VED increases (Figure 7b). This occurrence of a shifted diffraction angle can be attributed to the significant evaporation of Mn, with a lower boiling point compared to other elements in the FeMnCoCr HEA system, leading to a significantly lower elemental concentration at different volumetric energy densities. Additionally, due to its larger atomic diameter compared to Fe, Co, and Cr, the volatilization of Mn results in decreased lattice parameters within this HEA [21]. The diffraction pattern in Figure 7c exclusively demonstrates characteristic peaks corresponding to the austenitic crystal structure of Fe_50_Mn_30_Co_10_Cr_10_ powders, and the samples fabricated with different VEDs reveal the presence of martensite and austenite phase. Figure 7d demonstrates that the diffraction angles for peak (111) did not change as the VED increased except for the powder, and compared to the Fe_30_Mn_50_Co_10_Cr_10_ sample, its diffraction peak angle shows a more stabilized state at different VEDs. In summary, decreasing the Mn content from 50 to 30 at% transformed the lattice structure of the Fe_80−*x*_Mn*_x_*Co_10_Cr_10_ HEA from FCC single-phase to FCC/HCP dual-phase. Gradual heat accumulation during the LPBF process results in a reduced temperature gradient and cooling rate in the melt pool, thereby promoting a thermotropic martensite phase transition. Notably, an increased intensity in the diffraction peak (220) was observed for the LPBF-printed Fe_30_Mn_50_Co_10_Cr_10_ and Fe_50_Mn_30_Co_10_Cr_10_ HEA samples when increasing the VED, indicating a preferential grain orientation within these samples.

**Figure 8:**
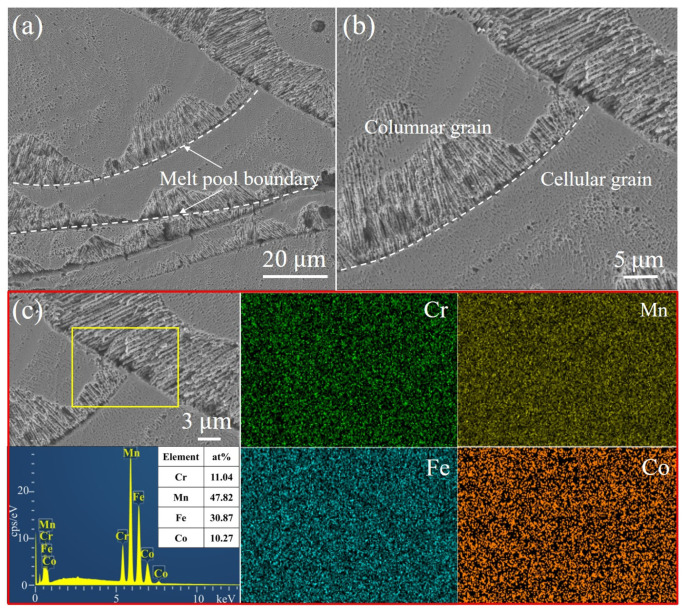
Microstructure and element distribution of the LPBF-printed Fe_30_Mn_50_Co_10_Cr_10_ HEA sample using a VED of 105.82 J·mm^−3^: (**a**) SEM image, (**b**) high-magnification image of (**a**), and (**c**) EDS mapping of element distributions.

The microstructure of the Fe_30_Mn_50_Co_10_Cr_10_ HEA sample printed using a volumetric energy density of 105.82 J·mm^−3^ reveals the presence of corrosion pits at the melt pool boundary, as depicted in Figure 8a,b. The accelerated corrosion rate observed at this boundary can be attributed to the existence of significant thermal stresses. Within the melt pool, columnar crystal structures grew along the heat flow direction at both the bottom boundary and inner area, while a cellular crystal structure was observed at the top region. Additionally, limited spherical pores were detected within the melt pool, which may have resulted from the volatilization of low-melting-point elements such as Mn during the LPBF process. Elemental mapping analysis depicted in Figure 8c demonstrates the uniform distribution of Fe, Mn, Co, and Cr elements within the Fe_30_Mn_50_Co_10_Cr_10_ HEA corresponding to locations where corrosion pits occurred at the melt pool boundary (highlighted by the yellow box), without any significant elemental segregation.

**Figure 9:**
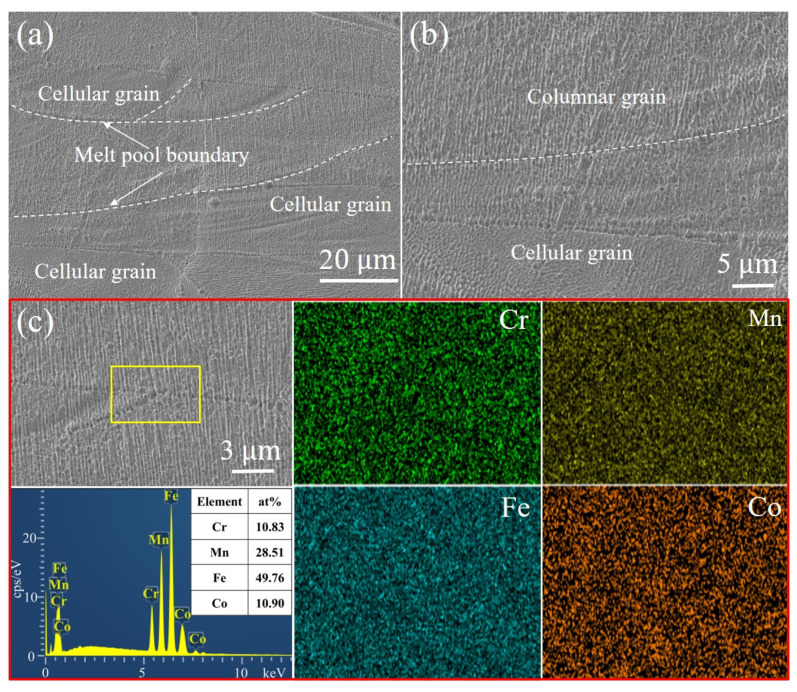
Microstructure and element distribution of the LPBF-printed Fe_50_Mn_30_Co_10_Cr_10_ HEA sample using a VED of 105.82 J·mm^−3^: (**a**) SEM image, (**b**) high-magnification image of (**a**), and (**c**) EDS mapping of element distribution.

The microstructure of the LPBF-printed Fe_50_Mn_30_Co_10_Cr_10_ HEA using an identical VED to Fe_30_Mn_50_Co_10_Cr_10_ is presented in Figure 9, showing primarily columnar and cellular grains with a distinct melt pool boundary. The morphology of the melt pool exhibited a semi-elliptical shape due to the Gaussian beam profile of the laser. Additionally, a significant heat accumulation led to large temperature gradients between solidified layers, facilitating the growth of columnar grains perpendicular or at an angle to the melt pool boundary. Numerous columnar grains extended across multiple solidified layers, while some gradually transformed into cellular grains as they propagated from perpendicular to the melt pool boundary to the direction of the heat flow. Figure 9c demonstrates a homogeneous distribution of HEA elements throughout the cross-section of the printed sample without any noticeable element segregation, except for minimal volatilization observed for Mn.

**Figure 10:**
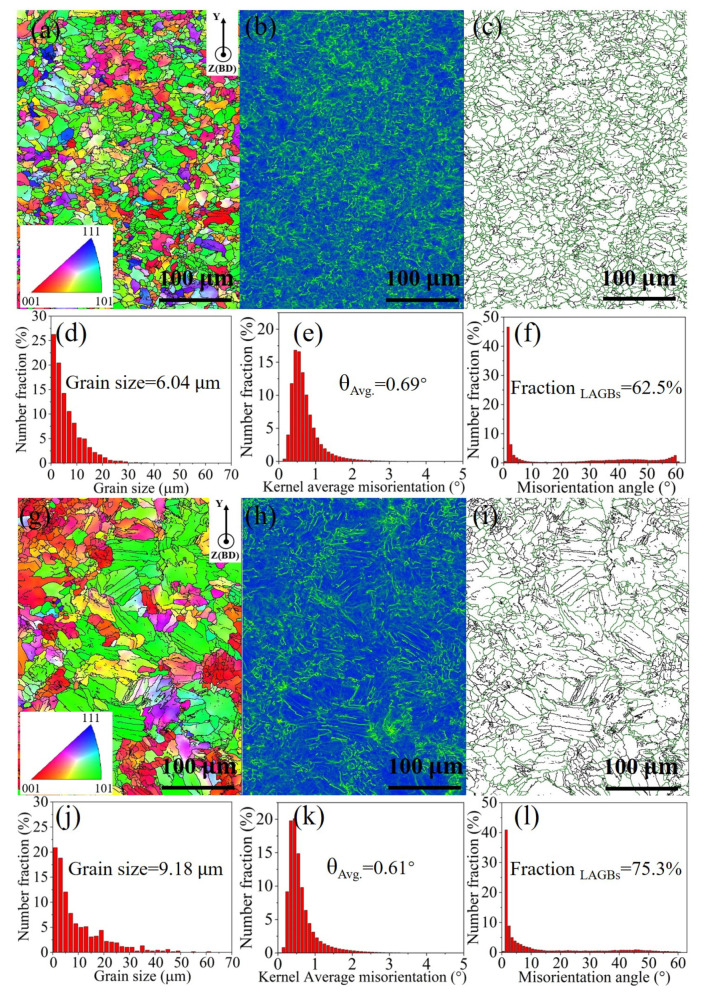
EBSD analyses of the Fe_80−*x*_Mn*_x_*Co_10_Cr_10_ HEA samples: (**a**–**c**) IPF, KAM color map, and misorientation map of the Fe_30_Mn_50_Co_10_Cr_10_ sample, respectively; (**d**–**f**) its grain size distribution, KAM distribution, and misorientation angle distribution, respectively. (**g**–**i**) IPF, KAM color map, and misorientation map of the Fe_50_Mn_30_Co_10_Cr_10_ sample, respectively, and (**j**–**l**) its grain size distribution, KAM distribution, and misorientation angle distribution, respectively.

To further investigate the microstructure of the LPBF-printed Fe_80−*x*_Mn*_x_*Co_10_Cr_10_ HEAs with different compositions in terms of grain size, crystal orientation, and grain texture, representative samples printed with the same VED (105.82 J·mm^−3^) were selected for microstructure analysis using EBSD (Figure 10). The Fe_30_Mn_50_Co_10_Cr_10_ HEA sample exhibits a smaller grain size compared to the Fe_50_Mn_30_Co_10_Cr_10_ sample (Figure 10a,g), with average grain sizes of 6.04 μm and 9.18 μm, respectively (Figure 10d,j). According to the Hall–Petch theory [35], increasing grain size is generally detrimental to the mechanical properties of metallic materials. A comparison between the KAM color map and KAM distributions reveals that the Fe_30_Mn_50_Co_10_Cr_10_ sample has a higher KAM value (0.69°) than the Fe_50_Mn_30_Co_10_Cr_10_ sample (0.61°) (Figure 10b,e,h,k), exhibiting a higher value of geometrically necessary dislocations (GNDs) in the Fe_30_Mn_50_Co_10_Cr_10_ HEA. Figure 10c,f,i,l demonstrate that the content of low-angle grain boundaries (LAGBs), ranging from 2° to 15° in orientation angles, is lower in the Fe_30_Mn_50_Co_10_Cr_10_ sample (62.5%) compared to that in the Fe_50_Mn_30_Co_10_Cr_10_ sample (75.3%). The reduction in the LAGBs contributes to improvements in mechanical properties due to their significant advantage as nucleation sites during creep and fatigue processes [36].

**Figure 11:**
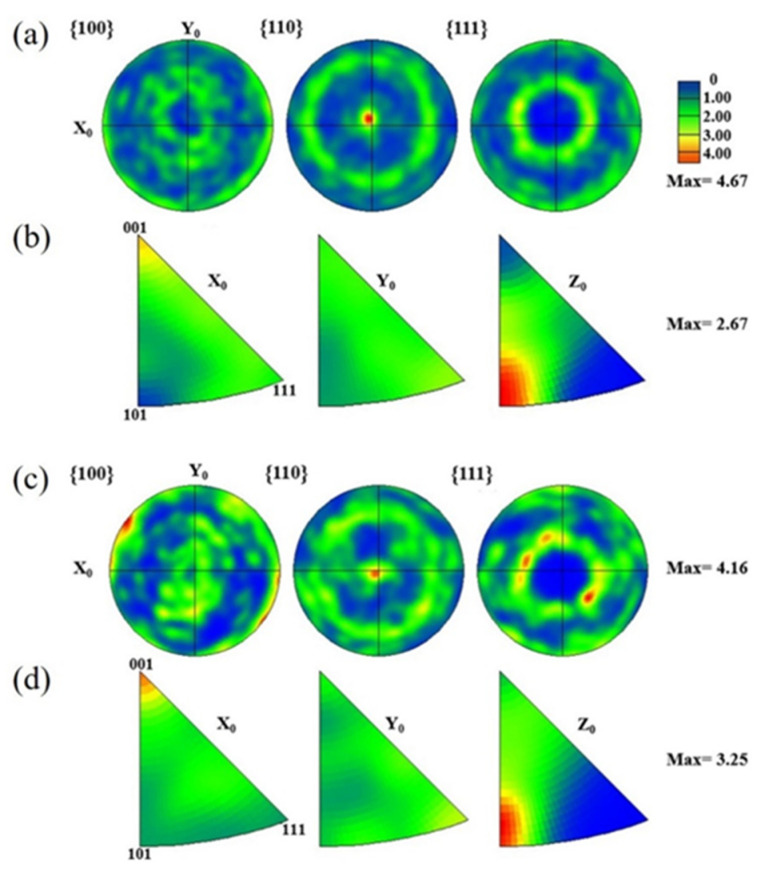
Grain orientation of LPBF-printed Fe_80−*x*_Mn*_x_*Co_10_Cr_10_ HEAs: (**a**,**b**) polar figure and IPF of the Fe_30_Mn_50_Co_10_Cr_10_ alloy along the X-Y plane, respectively, and (**c**,**d**) polar figure and IPF of the Fe_50_Mn_30_Co_10_Cr_10_ alloy along the X-Y plane.

The Fe_50_Mn_30_Co_10_Cr_10_ sample exhibits a more disordered grain orientation, as depicted in Figure 11a,c, while both HEAs demonstrate the preferential growth of grains with an orientation parallel to <110>. This is evidenced by the prominent peak of intensity that was observed at the central region of the (110) polar figure, which corresponds well with the strong (220) diffraction peaks observed in the XRD pattern (Figure 7). In Figure 11b,d, both HEAs exhibit a strong texture with a <101> orientation along the build direction, thus highlighting the influence of direction of heat flow on the resulting texture characteristics. Typically, austenitic crystal structures tend to grow with a prioritized texture with <001> oriented along the build direction [37]. However, this preferred orientation and microstructure can be altered by adjusting the rotating angle of successive solidified layers.

### 3.3. Mechanical Properties

**Figure 12:**
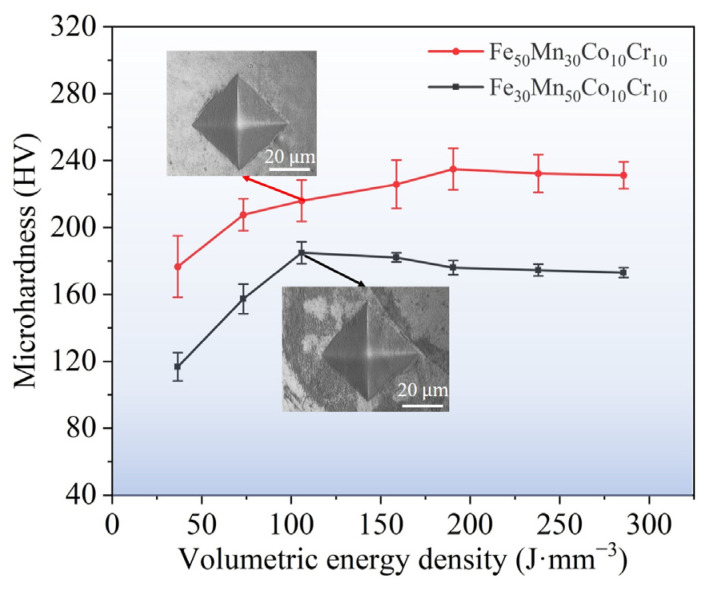
Microhardness of LPBF-printed Fe_80−*x*_Mn*_x_*Co_10_Cr_10_ HEA samples under different VEDs.

Figure 12 depicts the microhardness of as-printed Fe_80−*x*_Mn*_x_*Co_10_Cr_10_ HEAs with various VEDs. It is evident that with increasing VED, the microhardness of the Fe_50_Mn_30_Co_10_Cr_10_ samples and the Fe_30_Mn_50_Co_10_Cr_10_ samples tend to initially increase and then decrease. Moreover, the microhardness of Fe_50_Mn_30_Co_10_Cr_10_ remains consistently higher than that of Fe_30_Mn_50_Co_10_Cr_10_. In the case of a VED of 36.63 J·mm^−3^, the minimum microhardness values for the Fe_50_Mn_30_Co_10_Cr_10_ and Fe_30_Mn_50_Co_10_Cr_10_ samples are 176.66 HV and 116.74 HV, respectively. Furthermore, with the VED increasing to 105.82 J·mm^−3^, there is an increase in maximum microhardness in the Fe_30_Mn_50_Co_10_Cr_10_ sample, reaching up to 184.98 HV; however, this increase in microhardness is accompanied by a significant presence of microporous defects in samples at higher VEDs, leading to an overall decrease in microhardness. In the case of a VED of 190.47 J·mm^−3^, the maximum microhardness on the Fe_50_Mn_30_Co_10_Cr_10_ sample reaches 234.96 HV, due to martensitic ε-phase formation resulting in higher microhardness.

**Figure 13:**
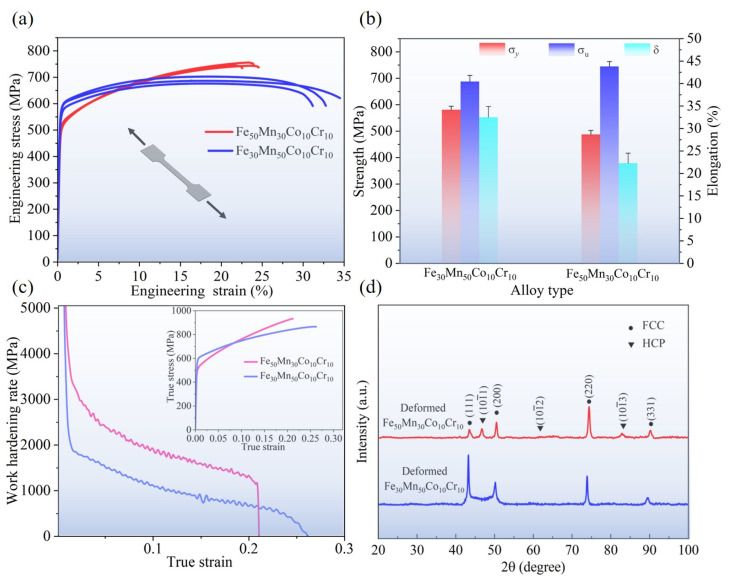
Tensile properties of LPBF-printed Fe_30_Mn_50_Co_10_Cr_10_ and Fe_50_Mn_30_Co_10_Cr_10_ HEAs under an identical VED of 105.82 J·mm^−3^: (**a**) stress–strain curves, (**b**) histogram of yield strength, ultimate tensile strength, and elongation, (**c**) responses between work hardening rate and true strain, and inserted image presents true stress–strain curves, and (**d**) XRD patterns of the deformed samples printed by VED of the two HEAs.

Representative stress–strain curves of Fe_30_Mn_50_Co_10_Cr_10_ and Fe_50_Mn_30_Co_10_Cr_10_ HEAs fabricated by LPBF at identical VED are illustrated in Figure 13a. The yield strength and ultimate tensile strength of the Fe_50_Mn_30_Co_10_Cr_10_ samples are 487.60 MPa and 744.90 MPa, respectively. In contrast, the Fe_30_Mn_50_Co_10_Cr_10_ samples exhibit an σ_y_ value of 580.65 MPa and an σ_u_ value of 687.70 MPa. In addition, it is noteworthy that the elongation of the Fe_50_Mn_30_Co_10_Cr_10_ sample (22.3%) is lower than that observed for the Fe_30_Mn_50_Co_10_Cr_10_ sample (32.5%), as presented in Table 1. Interestingly, despite possessing a slightly higher σ_y_ value due to its smaller grain size, grain-refined single-phase FCC-structured Fe_30_Mn_50_Co_10_Cr_10_ samples exhibit significantly lower σ_u_ values compared to dual-phase Fe_50_Mn_30_Co_10_Cr_10_ samples with various grain sizes. The discrepancy can be attributed to an increase in martensitic phase fraction and higher residual stresses within the austenitic phase matrix during the tensile deformation process [38], causing an earlier attainment of yield limit point for the Fe_50_Mn_30_Co_10_Cr_10_ HEA.

The Fe_50_Mn_30_Co_10_Cr_10_ sample displays a higher work hardening rate compared to the Fe_30_Mn_50_Co_10_Cr_10_ sample (Figure 13c, attributed to the occurrence of a martensitic phase transition in the high dislocation region of austenite. This phase transition impedes dislocation slip and results in a sustained higher work hardening rate. Conversely, the fracture behavior of the Fe_50_Mn_30_Co_10_Cr_10_ sample is characterized by an abrupt decline in work hardening rate, indicating a “hard and brittle” effect during late strain stages. Although the martensitic phase transformation improves the ultimate tensile strength of the Fe_50_Mn_30_Co_10_Cr_10_ sample, it adversely affects its ductility. The deformed Fe_50_Mn_30_Co_10_Cr_10_ HEA samples in Figure 13d exhibit the presence of both martensite and austenite phases, while the deformed Fe_30_Mn_50_Co_10_Cr_10_ HEA samples consist of a sole austenite single phase. In conclusion, this study highlights the excellent mechanical properties achieved through LPBF printing for both Fe_30_Mn_50_Co_10_Cr_10_ and Fe_50_Mn_30_Co_10_Cr_10_ HEAs.

**Figure 14:**
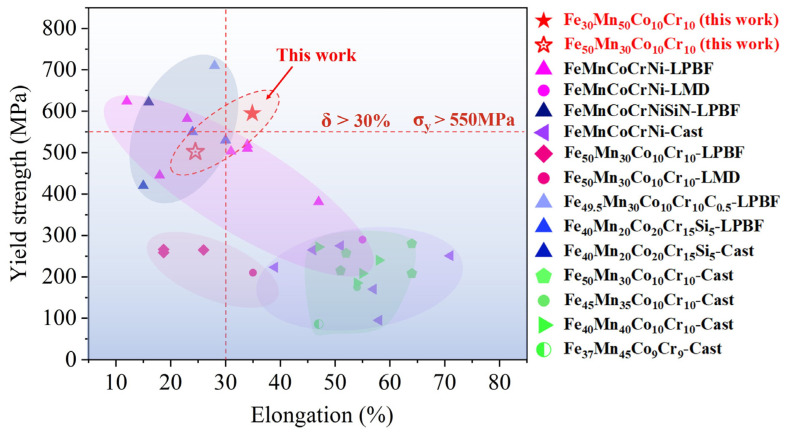
Comparison chart of the mechanical properties of the typical Fe_30_Mn_50_Co_10_Cr_10_, Fe_50_Mn_30_Co_10_Cr_10_ HEAs, and FeMnCoCr-based HEAs fabricated by AM [4,21,39,40,41,42,43,44,45,46,47,48,49,50] and traditional manufacturing process [9,10,11,12,13,14,15,16,17,18,29,50,51,52,53,54,55].

As shown in Figure 14, the LPBF-printed FeMnCoCr HEA systems exhibit an an exceptional combination of ductility and strength compared with previously reported additively manufactured FeMnCoCr HEAs [4,21,39,40,41,42,43,44,45,46,47,48,49,50] and cast FeMnCoCr-based HEAs [9,10,11,12,13,14,15,16,17,18,29,50,51,52,53,54,55]. This study shows that the elongation is over ~30% and the yield strength is over ~550 MPa. Meanwhile, the yield strength is significantly higher than the cast counterparts, and its ductility is comparable to pure and elementally added alloys.

**Figure 15:**
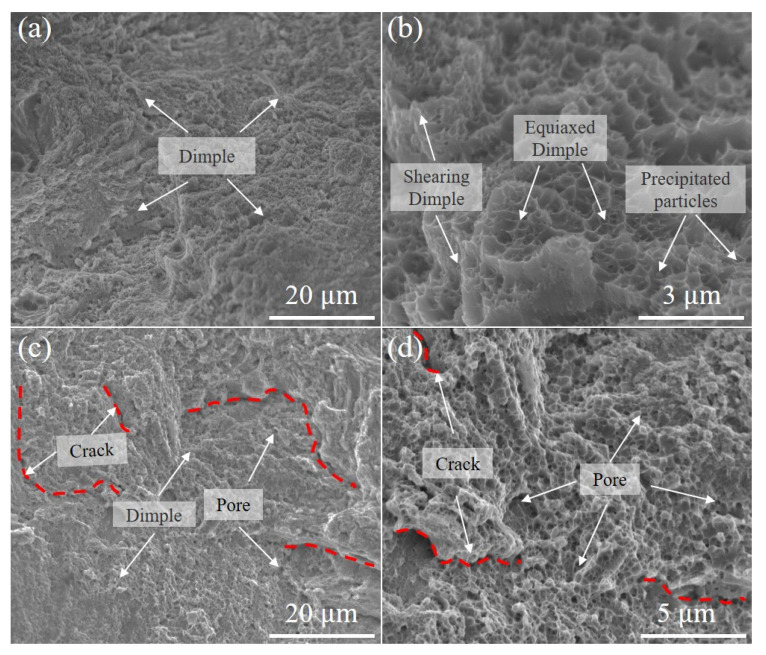
SEM images of fractured morphology of LPBF-fabricated Fe_80−*x*_Mn*_x_*Co_10_Cr_10_ samples after tensile deformation: (**a**) Fe_30_Mn_50_Co_10_Cr_10_ sample, (**b**) high-magnification image of (**a**), (**c**) Fe_50_Mn_30_Co_10_Cr_10_ sample, and (**d**) high-magnification image of (**c**). The red dotted lines represent crack.

The fracture surface of the Fe_30_Mn_50_Co_10_Cr_10_ sample exhibits a multitude of equiaxial and shear dimples, indicating typical ductile fracture behavior (Figure 15a,b). Additionally, sporadic precipitated particles are observed at the center of these dimples, contributing to stress concentration and promoting micro-void expansion during tensile deformation, thereby influencing the mechanical properties of the HEAs. Figure 15c,d demonstrate that the Fe_50_Mn_30_Co_10_Cr_10_ sample displays a characteristic ductile fracture with numerous dimples containing pore and crack defects. This can be attributed to the presence of heterogeneous interfacial stresses between austenite and newly formed martensite, which induce the initiation and propagation of microcracks and pores within the dual-phase structure, consequently resulting in reduced ductility.

**Figure 16:**
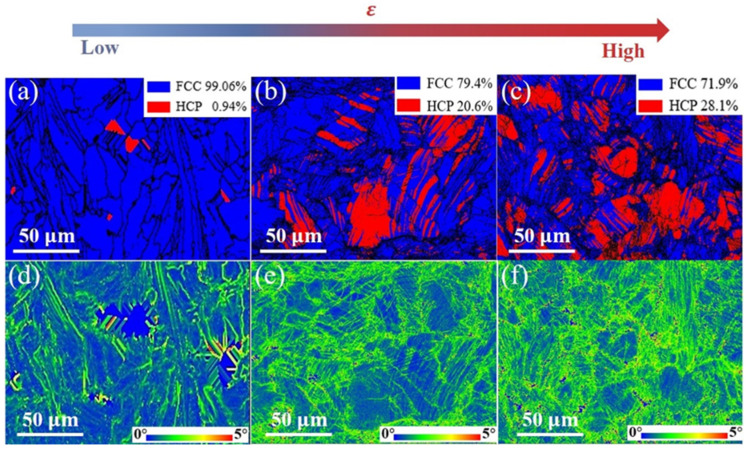
EBSD results illustrating the microstructural evolution in the Fe_50_Mn_30_Co_10_Cr_10_ HEA sample under increasing tensile strains: (**a**–**c**) phase distributions at strains of 0%, 10%, and 20%, respectively, and (**d**–**f**) KAM maps at strains of 0%, 10%, and 20%, respectively.

In summary, the two HEAs have extraordinary tensile properties, especially the characteristics (brittleness) of the Fe_50_Mn_30_Co_10_Cr_10_ HEA during tensile deformation. Moreover, the Fe_30_Mn_50_Co_10_Cr_10_ HEA has better ductility and does not present transition behavior during deformation (Figure 13d). Therefore, it is valuable to further explore the microstructural evolution of Fe_50_Mn_30_Co_10_Cr_10_ HEAs under different strains. The microstructure deformation in the LPBF-printed Fe_50_Mn_30_Co_10_Cr_10_ sample was investigated using EBSD at various regions of its fracture with different strain levels. The melt pools experienced significant temperature gradients and cooling rates during LPBF. The appearance of a minimal presence of HCP phase in the LPBF-fabricated Fe_50_Mn_30_Co_10_Cr_10_ HEA can be attributed to a non-equilibrium solidification of the melt pools significantly promoting a thermal martensitic transformation in this HEA (Figure 16a). With an increase in local strain from 0 to 20%, the average proportion of the HCP phase obtained from EBSD increases accordingly, ranging from 0.94% to 28.1% (Figure 16b,c). At the initial stage of deformation, regions with or neighboring to the FCC phase present significantly larger KAM values (Figure 16e), while regions with the HCP phase exhibit less noticeable KAM values. The KAM values indicate the density of GNDs to be within a specified area [56]; thus, larger KAM values in the FCC phase regions indicate a higher density of GNDs and pronounced plastic strains within these areas. These results illustrate that it is predominantly the FCC phase that accommodates plastic strain at this stage. Regarding strain partitioning behavior during the intermediate and late stages of deformation, the FCC phase regions still obtain larger KAM values, while most of the HCP phase regions exhibit even larger KAM values in comparison to those observed in the initial stage (Figure 16f). Generally, the HCP phase is known to be more rigid and less susceptible to deformation compared to FCC phase observed in most industrial alloys. Moreover, the GND at the FCC/HCP interface that contributes to the tendency of microporous generation is significantly higher compared with the other regions. This is an explanation for why it is more challenging for GND arrays to form within internal regions of the HCP-phase grains; instead, dislocations and stacking faults occur as slip bands within the HCP phase [57].

**Figure 17:**
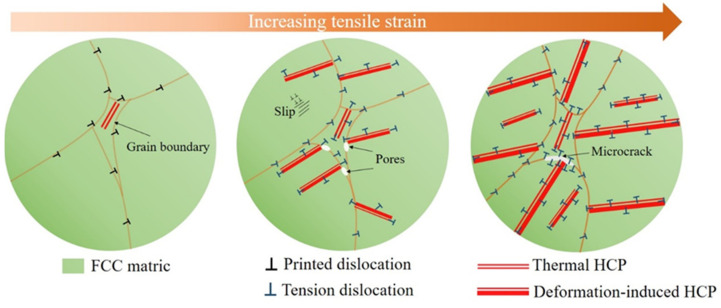
Schematic diagram of microstructural evolution of the Fe_50_Mn_30_Co_10_Cr_10_ HEA at increased strains.

The phase transformations in the Fe_50_Mn_30_Co_10_Cr_10_ HEA play a dual influence mechanism on its mechanical properties, as illustrated in Figure 17. During early deformation, the phase transformation enhances the strain hardening rate and the mechanical strength of the HEA. However, during late deformation stages, the dual-phase deformation of martensite and austenite causes a local high degree of incompatibility, leading to blocked dislocation movement and increased dislocation density near the interphase, generating stress concentration [29]. This promotes the nucleation of pores and the expansion of cracks. The formation of a martensite phase transition is more brittle, making it susceptible to induced crack expansion and damaging the HEA’s ductility. The Fe_50_Mn_30_Co_10_Cr_10_ HEA has lower stacking fault energy than the Fe_30_Mn_50_Co_10_Cr_10_ HEA [16], allowing for the easy bundling of incomplete dislocations to ensure cross slip as the stacking fault energy decreases. The synergistic deformation of these two phases generates a beneficial dynamic strain–stress partitioning effect [58]. During the deformation process, the GNDs accumulated at the interface of the dual-phase region generate heterogeneous deformation-induced stresses [59], which further lead to the plastic instability of the alloy. Therefore, the martensitic transformation plays a dual role in strength and ductility; i.e., one is useful for tensile strength and initial ductile elongation, while the other is harmful for ductility fracture. This contradicts the general view that the martensite phase transition is beneficial for ductility [60].

## 4. Conclusions

In this study, gas-atomized non-equimolar Fe_30_Mn_50_Co_10_Cr_10_ and Fe_50_Mn_30_Co_10_Cr_10_ powders were used for laser powder bed fusion. The microstructural evolution and mechanical properties of non-equimolar Fe_80−*x*_Mn*_x_*Co_10_Cr_10_ (*x* = 30%, 50%) HEAs fabricated by LPBF were comprehensively characterized. The findings are presented as follows.

The increased volumetric energy density led to a higher relative density for the LPBF-printed Fe_80−*x*_Mn*_x_*Co_10_Cr_10_ sample. At a VED of 75 and 85 J·mm^−3,^ the Fe_30_Mn_50_Co_10_Cr_10_ and Fe_50_Mn_30_Co_10_Cr_10_ samples achieved a relative density of 99%, respectively. Surface morphological observations revealed the formation of circular pores in the Fe_30_Mn_50_Co_10_Cr_10_ sample when Mn element evaporation occurred above a VED of 190 J·mm^−3^, resulting in a decrease in relative density.

The LPBF-printed and deformed Fe_30_Mn_50_Co_10_Cr_10_ samples exhibited single-phase FCC structures, but the Fe_50_Mn_30_Co_10_Cr_10_ samples exhibited dual-phase FCC-HCP structures under different VEDs. The diffraction peak intensities of both HEAs shifted with the increase in VED. The grain orientation of both HEAs demonstrated preferential growth of grains with an orientation parallel to <110>, which corresponds to the strong (220) diffraction peaks observed in the XRD pattern. The microstructure of both HEAs consisted of columnar and cellular grains. Moreover, with an increase in the strain of the Fe_50_Mn_30_Co_10_Cr_10_ sample from 0 to 20%, there was a corresponding rise in the average fraction of the martensitic phase range from 0.94% to 28.1%.

At a VED of 105.82 and 190.47 J·mm^−3^, the maximum microhardness of the Fe_30_Mn_50_Co_10_Cr_10_ and Fe_50_Mn_30_Co_10_Cr_10_ samples reached 184.98 HV and 234.96 HV, respectively. The yield strength, ultimate tensile strength, and elongation of the Fe_50_Mn_30_Co_10_Cr_10_ samples were 487.60 MPa, 744.90 MPa, and 22.3%, respectively. In contrast, the Fe_30_Mn_50_Co_10_Cr_10_ samples exhibited an σ_y_ value of 580.65 MPa, an σ_u_ value of 687.70 MPa, and an δ value of 32.5%. Despite the fact that the Fe_50_Mn_30_Co_10_Cr_10_ HEA possessed larger grains, lower dislocation density, and fewer LAGBs compared to the Fe_30_Mn_50_Co_10_Cr_10_ HEA in the horizontal plane, the ultimate tensile strength of the former was superior to that of the latter. Such an abnormal phenomenon can be attributed to the HCP phase formed during tensile deformation in the Fe_50_Mn_30_Co_10_Cr_10_ HEA, enhancing its mechanical strength. Moreover, the elongation of the Fe_50_Mn_30_Co_10_Cr_10_ HEA was lower compared with the Fe_30_Mn_50_Co_10_Cr_10_ HEA.

## Figures and Tables

**Table 1:** Tensile properties of Fe_80−*x*_Mn*_x_*Co_10_Cr_10_ alloy (at%).

HEA	σ_y_/(MPa)	σ_u_/(MPa)	δ/(%)
Fe_30_Mn_50_Co_10_Cr_10_	580.65 ± 13.48	687.70 ± 23.25	32.5 ± 2.4
Fe_50_Mn_30_Co_10_Cr_10_	487.60 ± 15.23	744.90 ± 18.47	22.3 ± 2.2

## Data Availability

The data presented in this paper are available on request from the corresponding author. The data are not publicly available due to privacy restrictions.
